# Metal Complexes of Macrocyclic Schiff-Base Ligand: Preparation, Characterisation, and Biological Activity

**DOI:** 10.1155/2013/289805

**Published:** 2013-07-01

**Authors:** Riyadh M. Ahmed, Enaam I. Yousif, Hasan A. Hasan, Mohamad J. Al-Jeboori

**Affiliations:** Department of Chemistry, College of Education, Ibn Al-Haitham, University of Baghdad, P.O. Box 4150, Adhamiyah, Baghdad, Iraq

## Abstract

A new macrocyclic multidentate Schiff-base ligand Na_4_L consisting of two submacrocyclic units (10,21-bis-iminomethyl-3,6,14,17-tricyclo[17.3.1.1^8,12^]tetracosa-1(23),2,6,8,10,12(24),13,17,19,21,-decaene-23,24-disodium) and its tetranuclear metal complexes with Mn(II), Co(II), Ni(II), Cu(II), and Zn(II) are reported. Na_4_L was prepared via a template approach, which is based on the condensation reaction of sodium 2,4,6-triformyl phenolate with ethylenediamine in mole ratios of 2 : 3. The tetranuclear macrocyclic-based complexes were prepared from the reaction of the corresponding metal chloride with the ligand. The mode of bonding and overall geometry of the compounds were determined through physicochemical and spectroscopic methods. These studies revealed tetrahedral geometries about Mn, Co, and Zn atoms. However, square planar geometries have been suggested for Ni^II^ and Cu^II^ complexes. Biological activity of the ligand and its metal complexes against Gram positive bacterial strain *Staphylococcus aureus* and Gram negative bacteria *Escherichia coli* revealed that the metal complexes become more potentially resistive to the microbial activities as compared to the free ligand. However, these metal complexes do not exhibit any effects on the activity of *Pseudomonas aeruginosa* bacteria. There is therefore no inhibition zone.

## 1. Introduction

Macrocyclic species based on transition metal compounds and multidentate ligands is an interesting field in chemistry and has been the subject of extensive research due to their potential applications in building block macrocyclic-based chemistry [1, 2] and environmental chemistry [3] and biomedical [4]. The chemistry of Schiff-base is an important field in coordination chemistry [5]. This is due to their ability to react with a range of metal ions forming stable complexes which have applications in different fields [6, 7]. One interesting application in the field of coordination chemistry has been to investigate the use of Schiff-base ligands to develop phenoxo-bridged binuclear complexes with homometallic and/or heterometallic centres. Complexes based on Schiff-base ligands play important roles in biomedical [8, 9], biomimetic, and catalytic systems [10, 11] and in supporting liquid crystalline phases [12]. A number of Schiff-base complexes have been used as oxygen carriers to mimic complicated biological systems [7, 11]. Furthermore, metal complexes of chromium, manganese, nickel, copper, zinc, and ruthenium with a wide variety of Schiff-bases are active oxidants for stoichiometric conversion and have been used as catalysts for carbonylation, hydrogenation, hydroformylation, and epoxidation reactions [13, 14]. Recently, we reported the formation of polymeric chain assemblies of some phenoxo-bridged binuclear transition metal complexes with multidentate Schiff-base ligand, namely, sodium(E)-6,60-((1E,10E)-(ethane-1,2-diylbis(azan-1-yl-1-ylidene) bis(methan-1-yl-ylidene) bis-(4-methyl-2((E)(pyridine-2-ylmethylimino)methyl)phenolate) H_2_L. As part of our group to explore the use of multidentate Schiff-base ligands for the building blocks of macrocyclic compounds, we describe here the formation of macrocyclic species of some phenoxo-bridged tetranuclear transition metal ions with the new macrocyclic Schiff-base. The ligand was derived via a template approach in which ethylenediamine fragments facilitated the linkage of the two units (10,21-bis-iminomethyl-3,6,14,17-tricyclo[17.3.1.1^8,12^] tetracosa-1(23),2,6,8,10,12(24),13,17,19,21,-decaene-23,24-disodium).

## 2. Experimental

### 2.1. Materials and Methods

All reagents were commercially available and used without further purification. Solvents were distilled from appropriate drying agents immediately prior to use.

### 2.2. Physical Measurements

Melting points were obtained on a Buchi SMP-20 capillary melting point apparatus and are uncorrected. IR spectra were recorded as KBr discs using a Shimadzu 8400 FTIR spectrophotometer in the range 4000–400 cm^−1^. Electronic spectra of the prepared compounds were measured in the region 250–900 nm for 10^-3 ^M solutions in DMF at 25°C using a Shimadzu 160 spectrophotometer. ^1^H- and ^13^C-NMR spectra were acquired in DMSO-d_6_ solution using a Brucker AMX400 MHz spectrometer with tetramethylsilane (TMS) as an internal standard. Mass spectra obtained by positive fast atom bombardment (FAB) were recorded on a VG autospec micromass spectrometer. Elemental analyses (C, H, and N) were carried out on a Heraeus instrument (Vario EL). Metals were determined using a Shimadzu (A.A) 680 G atomic absorption spectrophotometer. Chloride was determined using potentiometer titration method on a (686-Titro processor-665Dosimat-Metrohm Swiss). Conductivity measurements were made with DMSO solutions using a PW 9526 digital conductivity meter, and room temperature magnetic moments were measured with a magnetic susceptibility balance (Johnson Matthey Catalytic System Division).

## 3. Synthesis

### 3.1. Preparation of the Precursor Sodium (2,4,6-Triformyl Phenolate) (STFP)

 To a solution of p-hydroxybenzaldehyde (12.2 g, 10 mmol), hexamethylenetetramine (28.2 g, 20 mmol) in glacial acetic acid (50 mL), paraformaldehyde (30 g, 100 mmol) was added. The mixture was allowed to stir continuously until the deep orange viscous solution was obtained, and then heated up to 90°C for two hours. The solution was allowed to cool to room temperature, and then concentrated H_2_SO_4_ (10 mL) was carefully added. The resulting solution was refluxed for 30 min, and on treatment with distilled water (400 mL), a light orange precipitate was formed, which was stored overnight at 4°C. The orange product was isolated by filtration and washed in small amount of cold methanol to obtain *2,4,6-triformyl phenol (tfp)* [15]. The yielded product *(TFP)* (59%, 10.5 g) was mixed with equimolar amounts of NaOH (2.35 g, 5.8 mmol) in ethanol (25 mL). The mixture was allowed to stir for 30 min to give the sodium salt product (STFP) as a red-orange powder [16]. Yield: 65%, 7.66 g; m.p. 150°C. IR data (cm^−1^): 2995 (C–H) arom, 2924 and 2791 (C–H) aldehydic, 1683 (C=O), and 1240 (C–O).

### 3.2. Preparation of Na_4_L

 A solution of sodium *2,4,6-triformyl phenol*ate (STFP) (0.5 g, 2.4 mmol) in methanol (15 mL) was added slowly with stirring to a mixture of ethylenediamine (0.22 g, 3.6 mmol) dissolved in methanol (15 mL), and then 2–4 drops of glacial acetic acid were added to the reaction mixture. The mixture was allowed to reflux with stirring. After 4 h, the reaction was cooled to room temperature and then allowed to slowly evaporate to give a pale yellow viscous residue which was stirred for 30 min with a hot mixture of DMF/methanol 3 : 1 (15 mL). Solvent containing the required ligand was transferred to a vessel by decantation, and then solvent was removed under reduced pressure and residue was kept under vacuum for drying for 24 h. Yield: 0.77 g, 33%, m.p. = 178°C. IR data (cm^−1^): 3008 *ν*(C–H) arom, 2723 *ν*(C–H) iminic, 1632 and 1622 *ν*(C=N), 1350 *ν*(phenoxide). The ^1^H-NMR spectrum of the ligand in DMSO-d_6_ showed peaks at *δ*_H_ (400 MHz, DMSO-d_6_): 8.5 (8H, s, N=C–H); 8.3 (4H, s, N=C–H); 7.5 (8H, s, Ar–H); 4.5 (16H, m, N–CH_2_); 3.1 (8H, m, N–C–H_2_) and at *δ*_c_ (100.63 MHz, DMSO-d_6_): 57.49 (CH_2_N=C); 59.73 (CH_2_N=C); 117.91 (Ar); 119.13 (Ar); 121.7 (Ar); 123.4 (Ar); 143.1 (Ar); 160.08 (C–O); 162.23 (C=N) imine; 164.01 (C=N) imine. The positive (FAB) mass spectrum of Na_4_L showed a peak at *m*/*z* 967.103 (10%) corresponding to (M+Na)^+^ and the following fragments; 854.56 (32%) [M-(Na_2_O+CH_2_CH_2_)]^+^, 788.90 (26%) [M-{(Na_2_O+CH_2_CH_2_)^+^(NaCH=NO)}]^+^, 728.14 (32%) [M-(Na_2_O+CH_2_CH_2_)+(NaCH=NO)+(NH_2_CH_2_CH_2_NH_2_)]^+^, 533.21 (100%) [M-(Na_2_O+CH_2_CH_2_)+(NaCH=NO)+(NH_2_CH_2_CH_2_NH_2_)+(C_10_H_10_NH–C=NNa)]^+^, 480.68 (29%) [M-(Na_2_O+CH_2_CH_2_)+(NaCH=NO)+(NH_2_CH_2_CH_2_NH_2_)+(C_10_H_10_NH–C=NNa)+(NaCH_2_NH_2_)]^+^.

### 3.3. General Synthesis of Complexes

A solution of the Schiff-base ligand (1 mmol) dissolved in a 3 : 1 mixture of DMF/MeOH (25 mL) was allowed to stir for 15 min. A methanolic solution (15 mL) of the metal(II) salt (4.1 mmol) was then added dropwise, (metal (II) salts are hydrated chloride; MCl2·XH2O (M = Mn^II^, X = 4; Co^II^, Ni^II^ and Cu^II^; X = 6, 6 and 2, respectively. Zinc chloride was no hydrated)). The reaction mixture was heated under N_2_ for 2 h on a water bath, resulting in the formation of a solid mass which was washed several times with hot methanol and then dried at room temperature. Elemental analysis data, colours and yields for the complexes are given in Table 1.

^1^H-NMR spectrum of [Zn^II^_4_(L)]Cl_4_ in DMSO-d_6_ showed peaks at *δ*_H_ (400 MHz, DMSO-d_6_): 3.8 (8H, m, N–C–H_2_), 5.2 (16 H, m, N–C–H_2_), 7.3 (8H, d, 9.8 Hz, Ar–H), 8.1 (4H, br, N=C–H), and 8.7 (8H, br, N=C–H).

### 3.4. Determination of Biological Activity

Bioactivities were investigated using agar-well diffusion method [17]. The wells were dug in the media with the help of a sterile metallic borer with centres at least 24 mm. Recommended concentration (100 **μ**L) of the test sample 1 mg/mL in DMSO was introduced in the respective wells. The plates were incubated immediately at 37°C for 20 hours. Activity was determined by measuring the diameter of zones showing complete inhibition (mm). To examine the role of DMSO in the biological screening, separate studies were conducted with the solutions alone of DMSO, which showed no activity against any bacterial strains. All these complexes were found to be potentially active against these bacterial strains, except for the strain of *Pseudomonas aeruginosa*.

## 4. Results and Discussion

### 4.1. Chemistry

**Scheme 1:**
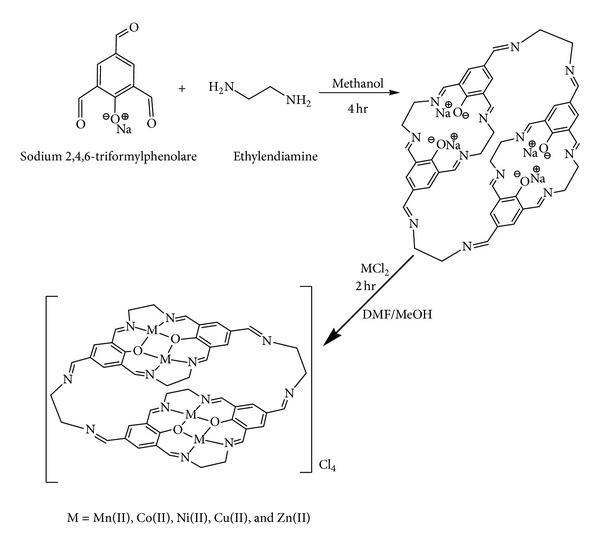
Synthesis scheme of the Schiff-base ligand Na_2_L and it's complexes.

A template approach was implemented to obtain the Schiff-base Na_4_L in a reasonable yield (Scheme 1). Using Na^+^ ion was found to be essential to form the ligand since otherwise only a polymeric mixture, partially soluble in hot DMF, was recovered via direct approach. The ligand was prepared from the reaction of sodium *2,4,6-triformyl phenol*ate (STFP) with ethylenediamine in mole ratios 2 : 3, respectively. The Schiff-base is soluble with stirring in DMF and DMSO but not in other common organic solvents. The ligand was characterised by elemental analysis (Table 1), IR (Table 2) and UV-Vis (Table 3) spectroscopy, and ^1^H- and ^13^C-NMR spectroscopy. The IR spectrum of the free Schiff-base shows characteristic bands at 1632, 1622, 1350, and 1031 cm^−1^ due to the *ν*(C=N), *ν*(phenoxide), and *ν*(C–O) functional groups, respectively. The UV-Vis spectrum of Na_4_L exhibits an intense absorption peak at 295 nm, assigned to *π* → π*. The peak at 322 nm assigned to *n* → π* transition. 

The bridged phenoxy tetranuclear complexes with Mn^II^, Co^II^, Ni^II^, Cu^II^ and Zn^II^ were synthesised by heating 1 mmole of the ligand with 4.1 mmole of the metal chloride in a mixture of DMF/MeOH. Complexes of general formula [M^II^_4_(L)]Cl_4_ ((M = Mn^II^, Co^II^, Ni^II^, Cu^II^, and Zn^II^) were obtained, Scheme 1). The complexes are air-stable solids, soluble in hot DMSO and DMF but not in other common organic solvents. The coordination geometries of the complexes were deduced from their spectra. The analytical data (Table 1) agree well with the suggested formulae. Conductivity measurements of Mn^II^, Co^II^, Ni^II^, Cu^II^, and Zn^II^ complexes in DMF lie in the 291.08–297.14 cm^2^ Ω^−1^ mol^−1^ range, indicating their 1 : 4 electrolytic behaviour (Table 1) [18].

### 4.2. FTIR and NMR Spectra

The most important infrared bands for the complexes together with their assignments are listed in Table 2. The IR spectra of the complexes exhibited ligand bands with the appropriate shifts due to complex formation. The *ν*(C=N) imine stretching band at 1632 cm^−1^ in the free Schiff-base is shifted to lower frequency and is observed at around 1589 cm^−1^ for the complexes. The bands are assigned to a *ν*(C=N) stretch of reduced bond order. This can be attributed to delocalisation of metal electron density (*t*_2*g*_) to the *π*-system of the ligand [19, 20], indicating coordination of nitrogen of the C=N moieties to the metal atoms [21]. In addition, the IR spectra of the complexes display peaks around 1620 cm^−1^, which may be attributed to the *ν*(C=N) imine stretching of the uncoordinated moieties. Further, bands in the region of 1518–1550 cm^−1^ in all the complexes suggest phenoxide bridging with the metal atoms [22, 23]. At lower frequency, the complexes exhibited bands around 619–688 and 516–584 cm^−1^, which could be assigned to *ν*(M–N) and *ν*(M–O) vibration modes, respectively [19, 24]. Due to the larger dipole moment change for M–O compared to M–N, the *ν*(M–O) usually appears at higher frequency than the *ν*(M–N) band [25]. The electronic spectra and magnetic moment data of the complexes are summarised in Table 3. 

The ^1^H-NMR spectrum in DMSO-d_6_ of the free Schiff-base shows peaks at 8.5 and 8.3 ppm assigned to –CH=N– (imine) protons, indicating that the azomethine protons are nonequivalent. In addition, the spectrum revealed two peaks around 4.5 and 3.7 ppm assigned to the CH_2_N fragment. The appearance of two chemical shifts may be because of the formation of two types of azomethine, (i) the one that is involved in the formation of the submacrocyclic component, and (ii) the one that facilitated the linkage between the two submacrocyclic parts. The ^13^C-NMR displays two peaks at ca. 59 ppm and two signals at ca. 162 ppm, indicating that the CH_2_N groups and the azomethine moieties are in a different environment. The NMR data is in accordance with the IR result in which two different peaks for C=N group were observed. The peak at 7.4 ppm is assigned to protons of aromatic ring. The ^1^H-NMR spectrum of [Zn^II^_4_(L)]Cl_4_ showed that the peaks of the azomethine protons are nonequivalent. Peaks observed around 8.7 ppm are related to the coordinated azomethine which are shifted slightly downfield, compared with those observed for the free ligand. A peak recorded at 8.1 ppm is attributed to the free azomethine groups (uncoordinated). The doublet at 7.3 ppm is assigned to protons of aromatic rings. The appearance of these protons as a doublet is due to mutual coupling and/or a fluctuation behaviour generated by (–CH_2_CH_2_–) moieties [15]. In general, the spectrum showed broader peaks compared with that for the free ligand. This may point out that a fluctuation behaviour occurred in DMSO solution.

### 4.3. Mass Spectra

The mass spectrum of the ligand was consistent with the proposed structural formula (Section 3). The positive ion FAB mass spectrum for [Cu^II^_4_(L)]Cl_4_ showed several peaks corresponding to successive fragmentation of the molecule. The mass spectrum of Cu(II) complex does not display a peak may refer to molecular ion peak. The first peak observed at *m*/*z* 1179 represents the molecular ion peak of the complex losing 2Cl moieties. Three distinct peaks were observed in the mass spectrum at *m*/*z* 1116, 932, and 902 which can be assigned to the fragments [M-{(2Cl+Cu+H)}]^+^, [M-(4Cl+Cu+(CH_2_CH_2_)_3_+N_2_)]^+^, and [M-(4Cl+Cu+(CH_2_CH_2_)_3_+N_2_+O_2_)+H]^+^, respectively. The FAB(+) mass spectrum for [Co^II^_4_(L)]Cl_4_ showed several peaks corresponding to successive fragmentation of the molecule. However, the spectrum failed to show a peak that refers to molecular ion peak. The first peak observed at *m*/*z* 1106 represents the molecular ion peak of the complex losing (2Cl+CH_2_CH_2_CN) fragment. Four distinct peaks were observed in the mass spectrum at *m*/*z* 897, 751, 518, and 328, can be assigned to the fragments [M-{(2Cl+CH_2_CH_2_CN)+(2Cl+Co+CH_2_CH_2_+(CN)_2_}]^+^, [M-{(2Cl+CH_2_CH_2_CN)+(2Cl+Co+CH_2_CH_2_+(CN)_2_+(CH_2_CH_2_)_2_+Co+O_2_)}]^+^, [M-{(2Cl+CH_2_CH_2_CN)+(2Cl+Co+CH_2_CH_2_+(CN)_2_+(CH_2_CH_2_)_2_+Co+O_2_)+(C_12_H_16_N_4_O)}]^+^, and [M-{(2Cl+CH_2_CH_2_CN)+(2Cl+Co+CH_2_CH_2_+(CN)_2_+(CH_2_CH_2_)_2_+Co+O_2_)+(C_12_H_16_N_4_O)+(CHNH)_2_+CH_2_CH_2_+2Co)}]^+^, respectively. The FAB (+) mass spectrum for [Ni^II^_4_(L)]Cl_4_ showed several peaks corresponding to successive fragmentations of the molecule. However, no peak related to molecular ion peak was detected in the spectrum. The first peak observed at *m*/*z* 1106 represents the molecular ion peak of the complex losing (2Cl+CH_2_CHCN)^+^ fragment. Five distinct peaks were observed in the mass spectrum at *m*/*z* 1031, 666, 638, 610, and 500, which can be assigned to the fragments [M-{(2Cl+CHCH_2_CN)+(NiO)}]^+^, [M-{(2Cl+CHCH_2_CN)+(NiO) + (C_14_H_13_N_4_Ni)}]^+^, [M-{(2Cl+CHCH_2_CN)+(NiO)+(C_14_H_13_N_4_Ni)+(CH_2_CH_2_)}]^+^, [M-{(2Cl+CHCH_2_CN)+(NiO)+(C_14_H_13_N_4_Ni)+(CH_2_CH_2_)+(CH_2_CH_2_)}]^+^, and [M-{(2Cl+CHCH_2_CN)+(NiO)+(C_14_H_13_N_4_Ni)+(CH_2_CH_2_)+(C_6_H_7_NO)}]^+^, respectively.

### 4.4. Electronic Spectra and Magnetic Moment Measurements

The electronic spectra of the complexes with the ligand exhibited various extents of hypsochromic shift of the bands related to the intraligand *π*→π* transition. The electronic spectrum of the tetranuclear-Mn(II) Schiff-base complex showed additional peaks at 318 and 423 nm assigned to the charge transfer (CT) and d–d transitions, respectively, in a distorted tetrahedral geometry [26, 27]. The observed magnetic moment for the Mn(II) complex 5.1 B.M is typical for tetrahedral geometry [19]. The electronic spectrum of the Co(II) complex is consistent with tetrahedral assignment [26, 28]. The spectrum of the Co(II) complex exhibited band characteristic of tetrahedral Co(II) complexes [26–29]. The magnetic moment was consistent with the tetrahedral environment around Co(II). The observed bands for the Ni(II) complex and its diamagnetic behaviour agrees well with the proposed square planar geometry [26, 30]. The electronic spectrum of the Cu(II) complex displays a broad band assigned to ^2^B_1_g → ^2^Eg transition, corresponding to square planar geometry [29, 30]. A magnetic moment of 1.51 B.M. is typical for four-coordinate copper complexes [31]. The spectrum of the Zn(II) complex exhibited bands assigned to ligand field *π*→π* and L → M charge transfer [26, 32]. The metal normally prefers tetrahedral geometry. The magnetic moment values for the tetranuclear macrocyclic complexes at RT are lower than the predicted values, indicating the presence of some antiferromagnetic interactions. This may occur from metal-metal interactions through the phenolic oxygen atoms and/or extensive electron delocalisation, which may be related to the formation of layer structures [15, 19, 33].

## 5. Biological Activity

The free Schiff-base macrocyclic ligand and its metal complexes were screened against *Staphylococcus aureus, Escherichia coli*, and* Pseudomonas aeruginosa *to assess their potential as an antimicrobial agent by disc diffusion method. The measured zone of inhibition against the growth of various microorganisms is listed in Table 4. It is found that the metal complexes have higher antimicrobial activity against Gram negative species only compared with the free ligand. Hence complexation increases the antimicrobial activity. Such increased activity of the metal complexes can also be explained on the basis of chelation theory [33]. According to this, the chelation reduces the polarity of the metal atom mainly because of the partial sharing of its positive charge with donor group and possible *π*-electron delocalisation over the whole ring. This increases the lipophilic character of the metal chelate system which favours its permeation through lipid layer of the cell membranes. 

## 6. Conclusion

In this paper, the synthesis and coordination chemistry of some macrocyclic-based tetranuclear metal complexes derived from the Schiff-base Na_4_L are investigated. A template approach was used to prepare the ligand in a reasonable yield. The complexes were prepared by mixing at reflux 1 mmole of the Schiff base with 4 mmole of the appropriate metal chloride. Tetranuclear complexes of the general formulae [M_4_(L)]Cl_4_ (where M = Mn, Co^II^, Ni^II^, Cu^II^, and Zn^II^) was obtained. Physicochemical analysis showed four cationic coordinate metal complexes were formed.

## Figures and Tables

**Table 1:** Colours, yields, elemental analyses, and molar conductance values.

Compound	Colour	Yield (%)	m.p.	Found (calcd.) (%)					Λ_*M*_ (cm^2^Ω^−1^mol^−1^)
				C	H	N	M	Cl	
Na_4_L	Pale yellow	47	178	60.4 (61.6)	4.52 (4.9)	17.7 (18.4)	—	—	
[Mn^II^_4_(L)]Cl_4_	Brown	44	285	46.6 (47.4)	3.4 (3.7)	13.6 (13.8)	17.6 (18.1)	10.7 (11.7)	294.01
[Co^II^_4_(L)]Cl_4_	Red-brown	50	320	46.2 (46.9)	3.4 (3.6)	13.4 (13.7)	18.1 (19.2)	10.9 (11.5)	295.58
[Ni^II^_4_(L)]Cl_4_	Green	46	305	46.36 (46.9)	3.33 (3.6)	13.39 (13.7)	18.14 (19.1)	10.88 (11.5)	292.71
[Cu^II^_4_(L)]Cl_4_	Green	42	270	45.9 (46.2)	3.3 (3.6)	13.2 (13.5)	19.8 (20.4)	10.9 (10.3)	291.08
[Zn^II^_4_(L)]Cl_4_	Yellow	42	265	44.86 (45.8)	3.4 (3.5)	13.2 (13.4)	19.9 (20.8)	10.1 (9.3)	297.14

**Table 2:** FTIR frequencies in (cm^−1^) of the compounds.

Compound	*ν*(C=N)_iminic_	*ν*(Phenoxide)	*ν*(M–N)	*ν*(M–O)
Na_4_L	1632, 1622	1350	—	—
[Mn^II^_4_(L)]Cl_4_	1575, 1618	1528	619	516
[Co^II^_4_(L)]Cl_4_	1579, 1620	1518	663	584
[Ni^II^_4_(L)]Cl_4_	1581, 1620	1525	688	565
[Cu^II^_4_(L)]Cl_4_	1577, 1617	1550	663	554
[Zn^II^_4_(L)]Cl_4_	1583, 1622	1540	632	555

**Table 3:** Magnetic moment and UV-Vis spectral data in DMF solutions.

Compound	*μ*_ eff_ (BM)	Band position (*λ* nm)	Extinction coefficient *ε*_max⁡_ (dm^3^ mol^−1^cm^−1^)	Assignments
Na_4_L		295	920	*π* → π*
		322	850	CT

[Mn^II^_4_(L)]Cl_4_	5.11	267	640	*π* → π*
		318	530	CT
		423	360	^ 6^A_1_g→^4^T_1_g

[Co^II^_4_(L)]Cl_4_	3.40	278	860	*π* → π*
		343	432	CT
		461	123	^ 4^T_1_g^(F)^→^4^T_1_g^(P)^

[Ni^II^_4_(L)]Cl_4_	0.02	271	758	*π* → π*
		316	410	CT
		664	87	^ 1^A_1_g→^1^A_2_g

[Cu^II^_4_(L)]Cl_4_	1.51	283	323	*π* → π*
		303	212	CT
		462	103	^ 2^B_1_g→^2^Eg

[Zn^II^_4_(L)]Cl_4_	Diamagnetic	291	574	*π* → π*
		311	1235	CT

**Table 4:** Biological activity for Schiff-base macrocyclic ligand and its complexes.

Compounds	*Staphylococcus aureus* (+)		*Escherichia coli* (−)		*Pseudomonas aeruginosa* (−)	
	5 mM	10 mM	5 mM	10 mM	5 mM	10 mM
Free ligand	−	+	−	+	−	−
[Mn^II^_4_(L)]Cl_4_ [Co^II^_4_(L)]Cl_4_	++	+++	+	++	−	−
[Ni^II^_4_(L)]Cl_4_	++	+++	+	++	−	−
[Cu^II^_4_(L)]Cl_4_	++	+++	+	++	−	−
[Zn^II^_4_(L)]Cl_4_	−	+	++	+++	−	−

(−): No inhibition/inactive, (+): (3–5) mm/active, (++): (6–8) mm/more active, (+++): (9–14) mm/highly active.
